# Deep Learning Models to Detect Anterior Cruciate Ligament Injury on MRI: A Comprehensive Review

**DOI:** 10.3390/diagnostics15060776

**Published:** 2025-03-19

**Authors:** Michele Mercurio, Federica Denami, Dimitra Melissaridou, Katia Corona, Simone Cerciello, Domenico Laganà, Giorgio Gasparini, Roberto Minici

**Affiliations:** 1 Department of Orthopaedic and Trauma Surgery, Magna Graecia University, “Renato Dulbecco” University Hospital, 88100 Catanzaro, Italy; michele.mercurio@unicz.it (M.M.); gasparini@unicz.it (G.G.); 2 Research Center on Musculoskeletal Health, MusculoSkeletal Health@UMG, Magna Graecia University, 88100 Catanzaro, Italy; 3 1st Department of Orthopaedic Surgery, National and Kapodistrian University of Athens, Attikon Hospital, 12462 Athens, Greece; dimitramelissaridi@gmail.com; 4 Department of Medicine and Health Sciences “Vincenzo Tiberio”, University of Molise, 86100 Campobasso, Italy; katia.corona@unimol.it; 5 School of Medicine, Saint Camillus University, 00131 Rome, Italy; simone.cerciello@unicamillus.org; 6 Department of Experimental and Clinical Medicine, Magna Graecia University, 88100 Catanzaro, Italy; domenico.lagana@unicz.it; 7 Radiology Unit, Department of Experimental and Clinical Medicine, Magna Graecia University, “Renato Dulbecco” University Hospital, 88100 Catanzaro, Italy; miniciroberto@gmail.com

**Keywords:** artificial intelligence, deep learning, knee, ligament, sport, anterior cruciate ligament, magnetic resonance imaging

## Abstract

Magnetic resonance imaging (MRI) is routinely used to confirm the suspected diagnosis of anterior cruciate ligament (ACL) injury. Recently, many studies explored the role of artificial intelligence (AI) and deep learning (DL), a sub-category of AI, in the musculoskeletal field and medical imaging. The aim of this study was to review the current applications of DL models to detect ACL injury on MRI, thus providing an updated and critical synthesis of the existing literature and identifying emerging trends and challenges in the field. A total of 23 relevant articles were identified and included in the review. Articles originated from 10 countries, with China having the most contributions (*n* = 9), followed by the United State of America (*n* = 4). Throughout the article, we analyzed the concept of DL in ACL tears and provided examples of how these tools can impact clinical practice and patient care. DL models for MRI detection of ACL injury reported high values of accuracy, especially helpful for less experienced clinicians. Time efficiency was also demonstrated. Overall, the deep learning models have proven to be a valid resource, although still requiring technological developments for implementation in daily practice.

## 1. Introduction

**Figure 1:**
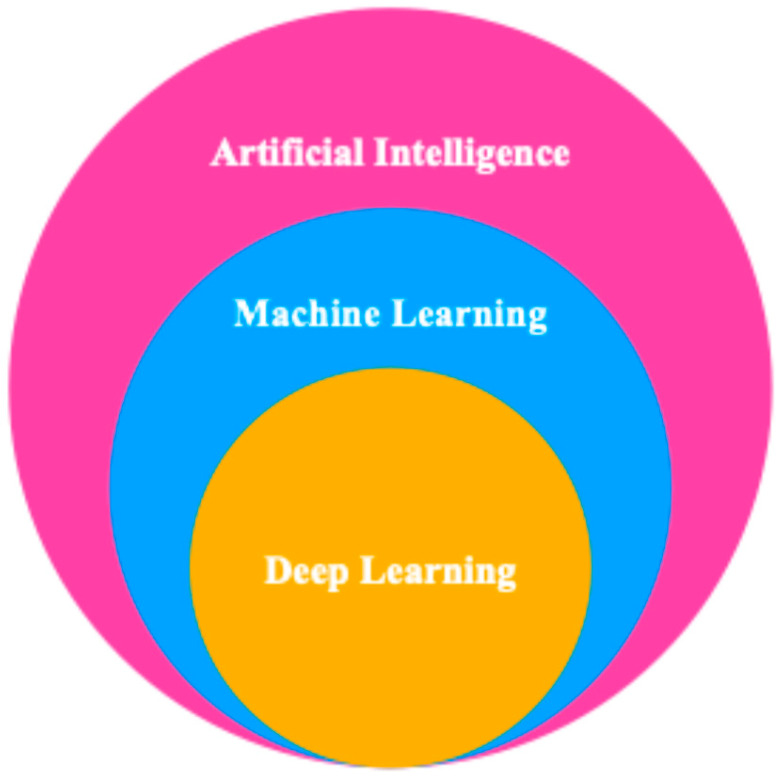
Machine learning and deep learning are subsets of artificial intelligence.

The anterior cruciate ligament (ACL) is the most injured ligament in the knee. Sports injuries are the main source of tears with a higher incidence among male subjects [1]. The annual reported incidence in the United States is 1 in 3500 people [2,3]. The symptoms include swelling, pain, knee deformation, and difficulty walking [4]. An ACL tear can range in severity from partial to complete, and each grade affects the knee’s stability and function differently [5]. A clinical exam, including Lachman and Pivot Shift tests, is essential for diagnosis [6]. Magnetic resonance imaging (MRI) is routinely used to confirm suspected diagnosis and to assess for concomitant injuries [7,8]. MRI presents benefits such as high contrast and resolution, non-invasiveness, and multi-planar imaging [9,10]. However, the current diagnosis process requires time-consuming examination by radiologists, which is also error-prone at scale. Indeed, a radiologist is required to examine each slice of the MRI scan, looking for ACL ruptures and other secondary complications, including bone marrow edema and anterior tibial translation [11]. Recently, numerous studies have investigated the role of artificial intelligence (AI) and its subgroups in the field of musculoskeletal system. AI refers to a branch of computer systems that are capable of performing tasks that mimic human cognitive functions such as learning and problem solving by analyzing and comparing data [12,13]. One specific area of investigation is how AI can contribute to the accurate diagnosis and characterization of ACL tears through imaging examinations to minimize medical errors. In this context, deep learning (DL), a sub-category of AI, is frequently used in medical imaging (Figure 1).

**Figure 2:**
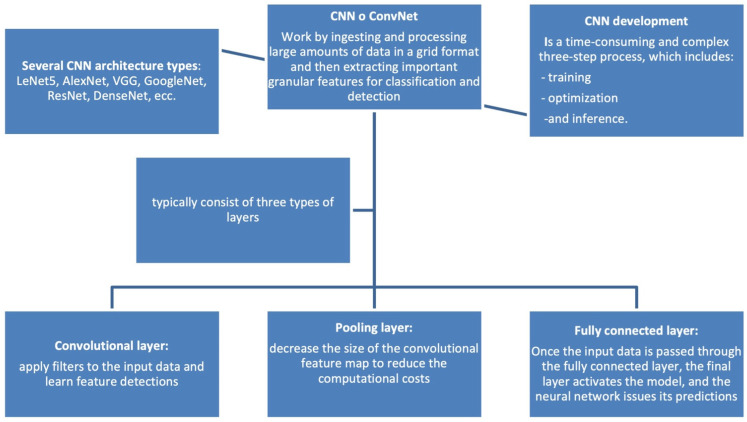
Convolutional Neural Network (CNN), also known as ConvNet. This is a specialized type of deep learning algorithm mainly designed for tasks that necessitate object recognition, including image classification, detection, and segmentation.

This tool consists of a series of inputs that pass through several interconnected layers of neurons that independently recognize different features and make predictions about large amounts of information [14,15]. Many DL target recognition systems are based on a convolutional neural network (CNN) because it can reduce the complexity of the overall network and training parameters and keep the data relatively constant in terms of scheduling, bias, and scaling (Figure 2).

Furthermore, the network structure is easy to train, optimize, and control [16]. To our knowledge, the use of DL models to detect ACL injury has been evaluated for a few years now, along with a few papers offering a comprehensive overview of this topic. Even though some reviews on DL models for detecting ACL injuries have been recently published [17], a new comprehensive review may be of significant value for several reasons. The field of AI, especially DL, is evolving rapidly, with the potential to transform musculoskeletal imaging [18]. A new review can include the latest studies capturing the most recent research and trends; evaluate newer models assessing the effectiveness of newer architectures like transformers or generative models; and consider larger and more diverse datasets, thus exploring how increased data availability and diversity impact model performance [19,20,21]. The aim of this study was to review the current applications of DL models to detect ACL injury on MRI, thus providing an updated and critical synthesis of the existing literature and identifying emerging trends and challenges in the field.

## 2. Materials and Methods

A review of the published literature was conducted and reported according to the Preferred Reporting Items for Systematic Reviews and Meta-Analyses statement [22]. The PubMed, MedLine, Scopus, and Cochrane Central databases were searched in December 2024. The search terms used to retrieve relevant articles were “deep learning”, AND “artificial intelligence”, AND “anterior cruciate ligament”, AND “MRI”, AND “diagnosis”. Only articles published over the last five years were considered. Two authors (FD and RM) independently reviewed the titles and abstracts to identify articles for inclusion in the database and contacted a third lead author (MM) in cases of major discrepancies. The reference list of each included article and the gray literature available at our institution were checked for possible additional articles. Other reviews, editorials, letters to the editor, and expert opinion papers were also considered but not included. The included articles are listed in Table 1.

A methodological quality assessment was independently conducted by 3 authors (FD, RM, and MM) with the modified Newcastle–Ottawa Quality Assessment Scale (Table 2). Substantial interobserver agreement (Cohen kappa coefficients ranging between 0.59 and 0.74) was reported. Based on the total score, the quality was classified as “low” (0–3), “medium” (4–6), and “high” (7–9). Criterion number: 1, representativeness of exposed cohort; 2, selection of unexposed cohort; 3, ascertainment of exposure; 4, evidence that the outcome of interest was not present at baseline; 5, comparability of cohorts based on design or analysis; 6, assessment of outcome; 7, follow-up period long enough to capture the results; 8, adequacy of the follow-up of the cohorts. Each study was scored with a maximum of one or two points for each numbered item within the categories, based on the rules of the modified Newcastle–Ottawa scale.

## 3. Results and Discussion

Although ACL injury can be assessed clinically, MRI is the best non-invasive imaging technique utilized to confirm the diagnosis. Moreover, a precise diagnosis is pivotal to planning and performing successful ACL treatment [43,44]. In the Results Section, we consider the applications of DL models to detect ACL lesions on MRI.

**Figure 3:**
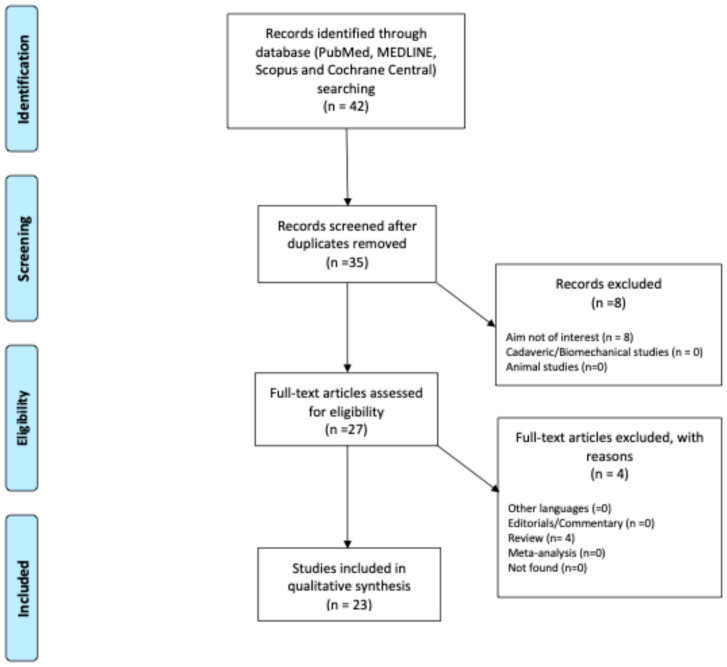
PRISMA flowchart.

A total of 42 relevant articles were identified through the initial search, resulting in 23 [5,7,9,16,23,24,25,26,27,28,29,30,31,32,33,34,35,36,37,38,40,41,42] studies that were included in the review (Figure 3).

The articles originated from 10 countries, with China having the most contributions (*n* = 9), followed by the United State of America (*n* =4), Japan (*n* = 2), Taiwan (*n* =2), and France, India, Vietnam, Korea, Taiwan, and Switzerland with 1 each.

All the studies only used CNN architectures in their methodology, not mentioning other methodologies like transformer-based and hybrid deep learning models. Specifically, only eight studies indicated the specific architecture type, as follows: MGACA, ResNet, DLCU-Net, CPDNN, VGGNet, SGNET, and DenseNet. All studies report the values of the accuracy and specificity of the models used, with values greater than 90%.

Another relevant element was the dataset used; the majority of the reports used an in-house dataset, with varying patient numbers, MRI images, MRI sequences, size images, and Tesla magnetic field (1.5 T vs. 3 T), while other studies used public dataset, such as the Chiba [27], Stanford (or MRNet or MRNetv-1.0) [45], and KneeMRI [46] datasets. For all studies, the examinations have been split into a training set, a validation set, and a hidden test set. The Chiba dataset include a total of 1177 MRI scans of knees collected from two institutions in Chiba, Japan, between 2014 and 2018. A 3.0 T MRI and a 1.5 T MRI l were used for Institute H and Institute K, respectively. The Stanford dataset, used in six papers, consists of 1370 knee MRI exams performed at Stanford University Medical Center. Examinations were performed using GE scanners, with a 3 T or a 1.5 T magnetic field. The KneeMRI is a publicly available dataset collected by Stajduhar et al. that contains 917 sagittal PD-weighted examinations acquired with a Siemens Avanto 1.5 T scanner at Clinical Hospital Centre Rijeka, Croatia, from 2007 until 2014 [46].

Furthermore, four studies compare the accuracy and specificity of CNN’s model between two MRI datasets, one in-house dataset and a public one, with comparable results.

Some authors, to mitigate constraints posed by limited dataset sizes, used domain adaptation methodologies and approaches such as data augmentation. Data augmentation [47] is a technique for artificially enlarging a training dataset by applying various transformations to the existing data. This technique is often used in machine learning and deep learning tasks, especially in the field of computer vision, to improve the generalization and robustness of the trained models. Common data augmentation techniques used are rotation, translation, scaling, flipping, shearing, zooming, brightness and contrast adjustment, and noise addition.

DL algorithms have been reported to improve the ability to diagnose cruciate ligament injuries using MRI. A first study applying AI to MRI [34] provided evidence that CNNs can be used to compare the diagnostic efficacy of different MRI sequences as follows: different fat-saturated (FS) and not-fat-saturated (NFS) images, including T1-weighted, T2-weighted, and STIR sequences, were considered. The results suggested that both FS and NFS sagittal images are suitable for the detection of ACL tears. The authors also emphasized that human variability is not a problem with a CNN, as the same result is expected every time for a given image set.

Shin et al. [35] created a CNN model for diagnosing ACL tears by using only one oblique sagittal image on which the largest ACL was observed, thus reporting a high diagnostic accuracy. Similarly, Minamoto et al. [32] evaluated the accuracy of a CNN system by a single sagittal image to compare the results with those of experienced human readers. One hundred sagittal MRI images from patients with and without ACL injuries were cropped and used for CNN training. The system demonstrated a sufficient capacity to define an ACL tear, with a higher specificity compared to human readers. Alternatively, other studies evaluated the role of the acquisition of multiple MRI slices. Chang et al. [7] used coronal MRI images of 260 subjects as input data as follows: 130 patients had ACL completely torn, whereas the other 130 had an intact ACL. The authors reported an accuracy of 96% of the CNN model to detect an ACL injury. Liu et al. [31] used whole T2-weighted fat-suppressed MRI images (coronal, sagittal, and axial planes) of 175 patients with complete ACL tear and 175 subjects with an intact ACL. The CNN model showed an area under the curve (AUC) of 0.98. Furthermore, several studies demonstrated that models constructed using multi-sequence MRI exhibited a significant performance advantage over their single-sequence counterparts [48,49,50].

Other investigators compared [26] the diagnostic performance of a DL model according to the magnetic field strength. The authors demonstrated that DL model sensitivity was comparable to that obtained by academic musculoskeletal radiologists (96% vs. 98%, respectively) at both 1.5 T and 3 T magnetic field strengths. However, the specificity of the DL model was significantly lower (93% vs. 100%, respectively). Furthermore, the subgroup analysis showed that the performance of the DL model decreased if MRI examinations were from outside institutions. An additional study, which developed a DL model based on a training dataset from 5 different centers, yielded an AUC of 0.99 and a sensitivity and specificity of 95.1% for both. The AI assistance significantly improved the accuracy of all clinicians, exceeding 96%. The most significant improvements in accuracy and time efficiency were reported for clinicians with moderately limited diagnostic expertise [37].

Furthermore, several studies reported application of 2D and 3D CNN models. The performance of a 3D CNN compared to a 2D CNN model was also investigated. The 2D convolutional kernels can use context about the height and width of the slice to make predictions. However, since 2D CNNs use a single slice as input, they are inherently unable to use the context from adjacent slices. Three-dimensional CNNs address this problem, improved the performance, but comes at a computational cost due to the higher number of parameters used. Despite this, several studies showed no clear difference in accuracy between the two methods. Namiri et al. [33] presented a fully automated ACL segmentation and a classification framework that provided hierarchical severity staging of ACL injuries. Notably, a higher accuracy was observed with the 2D model. Both the 2D and 3D architectures displayed a relatively high degree of sensitivity and specificity for intact, fully torn, and reconstructed ACLs. Similarly, other authors showed that 3D DL-based diagnosis systems yielded comparable results to 2D models [29]. Jeon et al. [27] designed a 3D CNN model to automatically extract relevant features, focusing on key regions where ACL tears are likely to occur. The model demonstrated good performance in the diagnosis of ACL tears, with accuracy comparable to expert radiologists. Furthermore, the authors highlighted that the specific lightweight nature of the model allowed it to be used in settings with limited computational resources, such as mobile devices or clinics with less powerful hardware. Awan et al. [5] proposed a further method for the localization of a knee ACL injury region called multi-scale guided attention-based context aggregation (MGACA). The authors demonstrated the superiority of the proposed MGACA method in comparison to other localization models in terms of overfitting, underfitting, and generalization.

In the most recently published paper included in the current review [40], the authors proposed a DL model consisting of three main modules as follows: a Dual-Scale Data Augmentation module to enrich the training data on both the spatial and layer scales; a selective group attention module to capture relationships across the layer, channel, and space scales; and a fusion module to explore the inter-relationships among various perspectives to achieve the final classification. Results demonstrated the superior performance of the proposed SGNET model in ACL tear detection compared to the MRNet [45], DLD [36], ELNet [51], VIT [52], and Med3D [53] models, achieving an accuracy of 0.925, a sensitivity of 0.926, a specificity of 0.924, and an AUC of 0.975.

Traditional CNNs have demonstrated significant potential in recognizing patterns in images, such as ACL lesions. These networks can learn hierarchical features from raw images without requiring the manual extraction of specific characteristics like ligament continuity or the presence of bone marrow edema. The ability to perform end-to-end learning has simplified the diagnostic process and reduced inter-observer variability. A notable limitation of traditional CNNs is their difficulty in handling small datasets, which is common in medical imaging [42]. Overfitting is a common issue in such cases, where the model tends to memorize specific details from the training data without effectively generalizing to new examples. Additionally, CNNs are often criticized for their black-box nature, meaning that the decision-making process of the model is not easily interpretable [32]. This lack of transparency can be a significant barrier to clinical adoption, where understanding the rationale behind the model’s diagnosis is crucial.

VGGNet, with its deep architecture consisting of 3 × 3 convolutional layers and 2 × 2 pooling operations, provides a simple yet effective approach for medical image analysis [35]. The most common versions are VGG16 and VGG19, which have 16 and 19 layers, respectively. Transfer learning using pre-trained models from large datasets like ImageNet allowed for the adaptation of VGGNet to detect ACL lesions, significantly improving performance on smaller datasets [30]. Among its advantages, it is easy to implement and adapt to different classification tasks. This works well with moderately sized datasets. Despite its advantages, VGGNet is computationally expensive due to the large number of parameters, making it challenging to implement in real-world clinical settings. Additionally, the model is prone to overfitting on small datasets, which requires techniques like data augmentation or transfer learning from more generalized datasets to improve robustness. Futhermore, VGGNet’s depth and number of parameters make it a network that requires a lot of memory and computational power. Compared to more modern networks, such as ResNet, it is less efficient [30].

ResNet introduced the concept of residual connections, allowing networks to be extremely deep without suffering from the issue of vanishing gradients. This is particularly beneficial for ACL lesion diagnosis, where complex tissue patterns require significant depth to be effectively captured [23]. The ability to build very deep networks without compromising performance has led to improved accuracy in detecting even partial lesions. Among its advantages, it allows us to train deep networks without vanishing gradient problems, and it has excellent performance in computer vision competitions. However, ResNet still requires significant computational resources, especially with deeper models like ResNet-50 or ResNet-101, and it may not be suitable for environments with limited hardware [9]. Furthermore, like other deep learning models, ResNet requires large annotated datasets to achieve optimal performance, a critical factor given the limited availability of clinical datasets for ACL lesions.

DenseNet employs dense connections between layers, which enhances gradient flow and promotes feature reuse. This is particularly useful for extracting fine-grained details, which are crucial for identifying small ACL lesions in MRI scans [42]. Furthermore, DenseNet is parameter-efficient, reducing the overall number of parameters compared to networks such as VGGNet. Among the advantages, it reduces the number of parameters needed without compromising the capacity of the network and improves generalization and feature retrieval. Despite its efficiency in terms of parameters, DenseNet is still computationally intensive, especially when used with deeper models. Memory consumption during both training and inference can be a challenge in environments with limited resources [42].

The choice of the most suitable CNN model for ACL lesion diagnosis depends on several factors, including the availability of computational resources, the size of the dataset, and the complexity of the lesions. Deep architectures such as ResNet and DenseNet offer excellent lesion detection capabilities, especially for complex or partial injuries, but they require significant computational power.

While a growing body of studies has investigated the applicability of DL models to the MRI detection of ACL injuries, these applications are still primarily limited to the experimental realm. Joshi et al. [28] showed limitations related to training and testing time, performance on imbalanced hyperparameter tuning, computational complexity, generalization capability, and dataset size. Indeed, one of the big limitations in training CNN models is data scarcity in medical imaging. Approaches such as data augmentation (technique for creating new samples from the existing data and introducing additional variations) and synthetic data generation (artificially generated data, such as simulation, generative models, or data generation algorithms, which can be used to supplement or replace real-world data in machine learning and other applications) to mitigate constraints posed by dataset sizes should be considered.

Limitations include class imbalance and variability difficulties associated with MRI scans across studies. Many ACL investigations employ different MRI machines, field strengths, and acquisition techniques, which could substantially affect model performance. Note that different authors use different datasets of varying sizes, which does not allow for standardization in comparing models.

In addition, almost all studies included in the current review analyzed datasets from multiple centers with varying MRI scan quality. Overall, the power of DL models cannot be overlooked; in this context, Liang et al. [9] tested a CNN-based MRI system for ACL injury using arthroscopy as a standard reference and obtained an AUC of 0.89.

Regarding clinical applicability, reports indicate that DL models may not accurately identify partial ACL tears and may struggle to distinguish between complete and partial tears. Another limitation is that AI technologies may prove disproportionately expensive upon introduction [54]. Furthermore, the introduction of AI may raise concerns and skepticism among some clinicians and patients, as it represents a significant departure from traditional practices. The ongoing research will also inevitably spark debate on whether experienced clinicians or AI offers greater reliability [55]. Moreover, widespread AI implementation has brought to the forefront crucial ethical considerations, including legislative and privacy concerns.

Prospectives in the use of CNNs for the diagnosis of ACL injuries are very promising, with several directions for development that could revolutionize the diagnosis and treatment of these injuries. Neural networks could evolve by making themselves capable of detecting even the smallest or earliest ACL injuries, which might escape traditional visual diagnosis. This would lead to earlier diagnosis, reducing the risk of complications and combined injurie. They may also be able to distinguish between partial and complete injuries, and even suggest possible treatments based on severity. Another aspect could be the automatic detection of associated meniscal injuries. CNNs could evolve to detect both injuries simultaneously, reducing the need for multiple diagnostic investigations [3].

Currently, CNNs are mainly used to analyze MRI, but there are prospects for integrating computed tomography (CT) [56], ultrasound, and X-rays as well. Integrating these different techniques into a single AI system could improve the accuracy of diagnosis and reduce dependence on a single imaging modality.

Training CNNs requires large datasets, which are expensive and difficult to obtain. Self-supervised learning techniques could be an important step toward reducing the need for datasets, allowing models to learn from huge amounts of unlabeled data, such as medical images. Another possibility is the use of transfer learning, where models pre-trained on other tasks (such as general image recognition) can be adapted to the specific problem of ACL diagnosis [54]. This could accelerate the adoption of CNNs in diagnosis and improve the ability of models to adapt to new contexts with less specific data.

CNNs could be integrated into clinical decision support platforms to assist physicians in the diagnostic process by automatically suggesting likely diagnoses based on MRI images. These systems could also suggest treatment plans, monitor lesion evolution, and personalize patient management.

CNNs could be used not only in the initial diagnosis but also in the continuous monitoring of the ACL injury during treatment and recovery [57,58]. For example, CNNs could analyze longitudinal (pre- and post-operative) MRI images to monitor the healing of the ligament and detect any complications or reinjuries. In addition, the use of CNNs could expand to the assessment of post-operative rehabilitation, allowing for the automatic assessment of the improvement or deterioration of the ligament during recovery. This would be useful for adapting the therapeutic plan in real time.

Neural networks could evolve into hybrid models, combining CNNs with other AI architectures, such as recurrent neural networks (RNNs) or transformers. These models could also be able to process the temporal sequences of images or clinical data (e.g., MRI images collected during different phases of treatment), allowing for a deeper and more dynamic understanding of the injury and its recovery. Generative Adversarial Networks (GANs) could be used to generate synthetic imaging data that improves model training by increasing the amount and variety of data without the need to collect new images.

Another emerging field could be the collaboration between CNNs and robotic surgery [59]. CNNs could analyze, in real time, the images obtained by robotic devices during ACL repair operations, helping surgeons to pinpoint the exact location of the tear and optimize the accuracy of the treatment. The use of augmented reality (AR) could also be integrated into CNNs to visualize the tears in 3D and help surgeons plan the surgery. CNNs could analyze the images in real time and overlay diagnostic information or treatment plans directly on the AR view of the area in question.

In the future, CNNs could evolve to consider not only medical images but also the genetic, physiological, or clinical data of the patients. This would allow for the personalization of ACL diagnosis and treatment, based on the individual characteristics of the patient (e.g., age, gender, physical activity, and medical history) [60]. CNN-based predictive models that estimate the likelihood of recurrence or development of post-operative complications may also emerge, with the aim of providing more targeted and tailored treatments for the patient.

The prospects of using CNNs for the diagnosis of ACL injuries are highly promising and destined to revolutionize medicine. Due to the integration with other emerging technologies and the continuous evolution of deep learning algorithms, the future of ACL diagnostics could be increasingly automated, precise, and accessible.

## 4. Conclusions

Deep learning models for the MRI detection of ACL injury reported high values of accuracy and specificity, which is especially helpful for less experienced clinicians. Time efficiency was also demonstrated. Overall, the deep learning models have proven to be a valid resource, though they still require technological developments for implementation in daily practice.

## Figures and Tables

**Table 1:** Included studies.

Authors and Year	Where	Journal	Method	Software	% Accuracy and % Specificity	Dataset
Awan et al. 2023 [4]	Pakistan	PeerJ Computer Science	MGACA	Python (version 9.3.12); Python Software Foundation, Wilmington, DE, USA	98%–NA	In-house dataset (15,265 images)
Awan et al. 2021 [23]	Pakistan	Diagnostics	ResNet-14 CNN	Python (version 3.6); Python Software Foundation, Wilmington, DE, USA	92–94%	In-house dataset (917 knees sagittal plane)
Chang et al. 2019 [7]	USA	Journal of Imaging Informatics in Medicine	CNN	Python (version 3.5; Python Software Foundation, Wilmington, DE, USA	96–100%	In-house dataset
Chen et al. 2022 [24]	Taiwan	JMIR AI.	CNN	Python (version 3.x); Python Software Foundation, Wilmington, Delaware, USA and PyTorch (version 1.1.x) Meta AI, Menlo Park, CA, USA	96–96%	In-house dataset (1000 cases)
Cheng et al. 2024 [16]	China	Journal of Orthopaedic Surgery and Research	SVM	Python (version 3.6); Python Software Foundation, Wilmington, DE, USA	93–93%	In-house dataset (526 patients)
Dung et al. 2023 [25]	Vietnam	Diagnostic and Interventional Imaging	DCLU-Net CNN	TensorFlow (Available online: https://www.tensorflow.org, Accessed on: 12 December 2024) Google, Mountain View, CA, USA PyTorch (Available online: https://pytorch.org, Accessed on: 12 December 2024): Meta AI, Menlo Park, CA, USA	90–89%	In-house dataset (expanded—247 patients)
Germann et al. 2020 [26]	Switzerland	Invest Radiol.	DCNN	TensorFlow (version 1.11) Google, Mountain View, CA, USA	AUC 93–93%	In-house dataset (5802 MRI)
Jeon et al. 2021 [27]	Japan	IEEE Journal of Biomedical and Health Informatics	CNN	PyTorch (Available online: https://pytorch.org, Accessed on: 12 December 2024): Meta AI, Menlo Park, CA, USA	AUC 98–95%	Chiba and Stanford datasets+ data augmentation
Joshi and Suganthi 2022 [28]	India	Diagnostics	CPDCNN	Python (version 3.x), Python Software Foundation Beaverton, OR, USA	96%–NA	Stanford dataset
Li et al. 2023 [29]	China	Frontiers in Bioengineering and Biotechnology	3D CNN	PyTorch (Available online: https://pytorch.org, Accessed on: 12 December 2024): Meta AI, Menlo Park, CA, USA	AUC 97% and 93% for each dataset–NA	In-house dataset + Stanford dataset
Li et al. 2021 [30]	China	Journal of Healthcare Engineering	pretrained VGG16	TensorFlow (Available online: https://www.tensorflow.org, Accessed on: 12 December 2024) Google, Mountain View, California, USA PyTorch (Available online: https://pytorch.org, Accessed on: 12 December 2024): Meta AI, Menlo Park, CA, USA	92–91%	In-house dataset (30 patients)
Liang et al. 2023 [9]	China	BMC Medical Imaging	Res-Net CNN	TensorFlow (Available online: https://www.tensorflow.org, Accessed on: 12 December 2024) Google, Mountain View, CA, USA PyTorch (Available online: https://pytorch.org, Accessed on: 12 December 2024): Meta AI, Menlo Park, CA, USA	81–65%	In-house dataset (468 images) + data augmentation
Liu et al. 2019 [31]	USA	Radiology: Artificial Intelligence	CNN	Python (version 2.7); Python Software Foundation, Wilmington, DE, USA	AUC 98–96%	In-house dataset (300 subjects)
Minamoto et al. 2022 [32]	Japan	BMC Musculoskeletal Disorders	CNN	Python (version 3.6.7); Python Software Foundation, Wilmington, DE, USA	88–86%	In-house dataset (100 epochs) + data augmentation
Namiri et al. 2020 [33]	USA	Radiology: Artificial Intelligence	2D e 3D CNN	TensorFlow (Available online: https://www.tensorflow.org, Accessed on: 12 December 2024) Google, Mountain View, CA, USA PyTorch (Available online: https://pytorch.org, Accessed on: 12 December 2024): Meta AI, Menlo Park, CA, USA	92% and 89–90% and 88%	In-house dataset (1243 knee MRI)
Richardson. 2021 [34]	USA	Current Problem in Diagnostic Radiology	CNN	Python (version 1.2.2); Python Software Foundation, Wilmington, DE, USA	99–99%	In-house dataset (2007 images)
Shin et al. 2022 [35]	Korea	Medicine	VGGNet model CNN	TensorFlow (Available online: https://www.tensorflow.org, Accessed on: 12 December 2024) Google, Mountain View, CA, USA PyTorch (Available online: https://pytorch.org, Accessed on: 12 December 2024): Meta AI, Menlo Park, CA, USA	94%–NA	In-house dataset (130 images)
Tran et al. 2022 [36]	France	European Radiology	CNN	TensorFlow (Available online: https://www.tensorflow.org, Accessed on: 12 December 2024) Google, Mountain View, CA, USA	AUC 94–91%	In-house dataset vs. two esternal dataset (Stanford dataset and KneeMRI)
Wang et al. 2024 [37]	China	Arthroscopy	CNN	TensorFlow (Available online: https://www.tensorflow.org, Accessed on: 12 December 2024) Google, Mountain View, CA, USA PyTorch (Available online: https://pytorch.org, Accessed on: 12 December 2024): Meta AI, Menlo Park, CA, USA	96–95%	Internal dataset (22,767 MRIs) vs. external validation dataset (4086 MRIs)
Wang et al. 2024 [38]	China	QIMS	CNN (YOLOv5m and ResNet-18)	PyTorch (version 1.11.0) Meta AI, Menlo Park, CA, USA	95–95%	OAI dataset [39] (1589 knees) vs. external (Stanford and kneeMRI dataset)
Wang et al. 2024 [40]	China	Tomography	SGNET model CNN	PyTorch (Available online: https://pytorch.org, Accessed on: 12 December 2024) Meta AI, Menlo Park, CA, USA	92–92%	Stanford dataset
Xue et al. 2024 [41]	China	Nature	U-Net CNN	Python (version 2.7); Python Software Foundation, Wilmington, DE, USA	99–97%	In-house dataset (862 participants)
Zhang et al. 2020 [42]	China	Journal of Magnetic Resonance Imaging	3D DenseNet CNN	ITK-SNAP software (v. 3.6; Available online: http://www.itksnap.org, Accessed on: 12 December 2024)	96–94%	In-house dataset (408 subjects) + data augmentation

**Table 2:** Quality assessment of included studies according to the modified Newcastle–Ottawa scale.

	Criteria								Total	Quality
	1	2	3	4	5	6	7	8		
Awan et al. (2021) [23]	1	1	1	1	1	1	1	1	8	High
Awan et al. (2023) [5]	1	1	1	1	1	1	1	0	7	High
Chang et al. (2019) [7]	1	1	1	1	1	1	0	1	7	High
Chen et al. (2022) [24]	1	1	1	1	1	1	1	1	8	High
Cheng et al. (2024) [16]	1	1	1	1	1	1	1	1	8	High
Dung et al. (2023) [25]	1	1	1	1	1	1	1	1	8	High
Germann et al. (2020) [26]	1	1	1	1	1	1	1	1	8	High
Jeon et al. (2021) [27]	1	1	1	1	1	1	1	1	8	High
Joshi, Suganthi (2022) [28]	1	1	1	1	1	1	1	1	8	High
Li et al. (2023) [29]	1	1	1	1	1	1	1	1	8	High
Li et al. (2021) [30]	1	1	1	1	1	1	1	1	8	High
Liang et al. (2023) [9]	1	1	1	1	1	1	1	1	8	High
Liu et al. (2019) [31]	1	1	1	1	1	1	1	1	8	High
Minamoto et al. (2022) [32]	1	1	1	1	1	1	0	1	7	High
Namiri et al. (2020) [33]	1	1	1	1	1	1	1	1	8	High
Richardson (2021) [34]	1	1	1	1	1	1	1	1	8	High
Shin et al. (2022) [35]	1	1	1	1	1	1	1	0	7	High
Tran et al. (2022) [36]	1	1	1	1	1	1	1	1	8	High
Wang et al. (2024) [38]	1	1	1	1	1	1	1	1	8	High
Wang et al. (2024) [40]	1	1	1	1	1	1	1	1	8	High
Wang et al. (2024) [37]	1	1	1	1	1	1	1	1	8	High
Xue et al. (2024) [41]	1	1	1	1	1	1	1	1	8	High
Zhang et al. (2020) [42]	1	1	1	1	1	1	1	1	8	High

## Data Availability

The data presented in this study are available upon reasonable request from the corresponding author.
